# Reliability and validity of the Kurdish version of the patient health questionnaire-15 in a trauma-affected population

**DOI:** 10.1186/s12888-026-08020-1

**Published:** 2026-03-31

**Authors:** Ibrahim Mohammed, Hataw Ahmed Sharif, Jan Kizilhan, Bushra Hasin, Sergi Papiol, Monika Ruebekeil, Urs Heilbronner, Thomas G. Schulze, Martin Hautzinger

**Affiliations:** 1 Eberhard Karls University of Tübingen, Tübingen, Germany; 2 Institute for Transcultural Health Science – Institute for Transcultural Health Research, DHBW Stuttgart, Germany; 3 University of Sulaimani College of Medicine, Kurdistan Region, Sulaymaniyah, Iraq; 4 Institute of Psychiatric Phenomics and Genomics (IPPG), LMU University Hospital, LMU Munich, Munich, Germany; 5 Department of Psychiatry and Behavioral Sciences, SUNY Upstate Medical University, Syracuse, NY USA; 6 Department of Psychiatry and Behavioral Sciences, Johns Hopkins University School of Medicine, Baltimore, MD USA; 7 German Center for Mental Health (DZPG), partner site Munich-Augsburg, Munich, Germany; 8 Klinische Psychologie und Psychotherapie, Eberhard Karls University of Tübingen, Tübingen, Germany

**Keywords:** Trauma, PHQ-15, Kurdistan, Translation, Psychometrics

## Abstract

**Background:**

Somatic symptoms are common among trauma-exposed populations and are often associated with persistent distress and functional impairment, highlighting the need for culturally appropriate assessment tools.

**Objective:**

This study aimed to translate and culturally adapt the Patient Health Questionnaire-15 (PHQ-15), a screening measure of somatic symptom burden, into Central Kurdish and assess its psychometric properties among survivors of chemical gas exposure.

**Methods:**

A total of 534 survivors were recruited through community and registry sources. After translation and cultural adaptation, item 4 and item 11 were removed due to cultural and gender-specific considerations and limited psychometric performance, resulting in a 13-item version. Factor structure was examined using confirmatory factor analysis (CFA) and exploratory structural equation modeling (ESEM). Measurement invariance across gender and age groups was evaluated using multi-group CFA. Internal consistency was assessed using McDonald’s omega. An exploratory network analysis (EBICglasso) examined conditional associations among symptoms.

**Results:**

Both a unidimensional CFA model (CFI = 0.988, RMSEA = 0.054) and a three-factor ESEM model (CFI = 0.992, RMSEA = 0.046) showed good model fit, with the ESEM structure providing a more differentiated representation of pain/fatigue, gastrointestinal, and cardiopulmonary domains. Measurement invariance testing supported full scalar invariance across gender, while partial metric invariance was observed across age groups. Internal consistency was acceptable (ω = 0.70–0.83). Network estimates demonstrated acceptable stability, with correlation stability coefficients of 0.59 for edge strength and 0.52 for bridge strength, and should be interpreted as exploratory.

**Conclusion:**

The culturally adapted 13-item Kurdish PHQ demonstrates acceptable reliability and initial evidence of construct validity in trauma-exposed survivors of chemical attacks. The instrument may be used as a screening tool for somatic symptom burden in this context, while further validation in broader Kurdish populations is recommended.

**Supplementary Information:**

The online version contains supplementary material available at 10.1186/s12888-026-08020-1.

## Introduction

Somatic symptoms are common among the general population. Most people experience mild, temporary bodily sensations that are considered normal [1]. In certain cases, however, these symptoms become chronic and distressing, leading to marked functional impairment and psychological distress. Somatic symptom disorder (SSD) as defined in the Diagnostic and Statistical Manual of Mental Disorders, Fifth Edition, Text Revision (DSM-5-TR) is a clinically significant condition defined by chronic physical symptoms associated with excessive thoughts, emotions, or behaviors related to these symptoms [2]. Affected individuals often report negative thoughts, increased negative emotions, and help-seeking behaviors. Persistent somatic symptoms are also strongly associated with psychological distress, particularly depressive and anxiety symptoms. Trauma exposure also increases vulnerability to both somatic and psychological symptoms [3].

Survivors of traumatic events frequently report chronic pain, fatigue, and sleep disturbances alongside post-traumatic stress symptoms. A previous study of Kurdish chemical attack survivors has demonstrated lasting physical and psychological consequences, including impaired quality of life, increased bodily pain, and reduced energy compared with non-exposed populations [4–7]. These combined effects highlight the need of culturally appropriate screening tools for trauma-affected populations.

Somatic symptoms create significant challenges not only for affected individuals but also for healthcare systems. They frequently lead to repeated medical consultations and unnecessary diagnostic procedures, contributing to personal suffering and economic cost. Therefore, it is essential to validate instruments such as the PHQ-15 across cultural and linguistic contexts, which may improve the assessment of somatic symptoms and inform clinical decision-making [8, 9].

The PHQ-15 is a widely used screening tool that measures somatic symptom burden and helps identify individuals who may require further evaluation rather than serving as a diagnostic instrument for SSD [9]. It assesses the frequency and severity of fifteen common physical symptoms. Factor-analytic studies consistently demonstrate a multidimensional structure, typically reflecting symptom domains related to pain, gastrointestinal disturbance, cardiopulmonary problems, and fatigue [10–13]. The instrument has been validated in diverse populations and settings, with strong evidence supporting its psychometric soundness [9].

Following cultural adaptation, the PHQ-15 was modified to a 13-item version to address cultural and gender-specific considerations among trauma-exposed survivors of chemical attacks. To examine the complex structure of somatic symptoms, we employed exploratory structural equation modeling (ESEM) [14, 15], which allows theoretically plausible cross-loadings, and network analysis, which conceptualizes symptoms as dynamically interconnected components of a broader system. This approach complements factor analyses by identifying potential ‘bridge’ symptoms that link different symptom domains, which may be important targets for clinical intervention or further research, especially in trauma-exposed populations where symptom interactions are complex [16]. These approaches provide insight into symptom patterns and relationships, complementing traditional latent factor analyses, without implying causal mechanisms.

By providing a culturally adapted and psychometrically evaluated 13-item Kurdish PHQ in trauma-exposed survivors of chemical attacks using ESEM and multi-group CFA, this study offers the first validation of the instrument in this population and presents evidence on its factor structure and symptom relationships.

The aims of the study are to validate the psychometric properties of the Kurdish PHQ-13 in the trauma-exposed survivors of chemical attacks in the Kurdistan region, including (1) examine the factor structure of the Kurdish PHQ-13 using both (CFA) and (ESEM), (2) evaluate measurement invariance across gender and age groups to assess the consistency and validity of the scale, (3) investigate associations between socio-demographic, clinical, and psychological factors and Kurdish PHQ-13 scores and (4) explore symptom interrelations using network analysis and identify bridge symptoms linking different somatic domains.

## Method and instruments

The validation of the Kurdish PHQ-13 was part of a larger study investigating factors influencing the development of depression, anxiety, and post-traumatic stress disorder among survivors of the Yazidi genocide and chemical attack. The study received ethical approval (see Ethics Approval and Consent to Participate section).

Data collected from multiple locations in the Kurdistan Region, Halabja (*n* = 373), Sulemani (*n* = 54), the Khormal (*n* = 44), the Serwan (*n* = 41), and the Tawella (*n* = 22) over seven months, from March to September 2023, resulting in a total of 534 participants from 679 contacted individuals, corresponding to a response rate of approximately 79%. Written informed consent was obtained from all enrolled participants. Additionally, 145 potential participants did not participate, 37 explicitly refused, while others were unreachable or deceased.

Participants were Kurdish survivors of chemical gas attacks, recruited through three pathways. The largest group (*n* = 353, 66%) was identified via official registries maintained by the Kurdistan Regional Government’s Ministry of Martyrs and Anfal Affairs, these registries document survivors eligible for financial compensation following formal evaluations. A second group (*n* = 102, 19%) comprised individuals with documented exposure and formal medical assessment who were not yet registered due to administrative delays. The remaining participants (*n* = 79, 15%) were recruited through snowball sampling, eligibility confirmed using structured trauma exposure scale (see Supplementary Table S1).

Kurdish PHQ-13 total scores were compared across the three recruitment pathways using one-way ANOVA to examine potential differences in symptom severity. No significant differences were observed, suggesting that recruitment source is unlikely to have substantially influenced reported symptom severity.

Inclusion criteria required confirmed exposure to chemical gas, informed consent to participate, and physical and mental capacity to complete assessments. Exclusion criteria included inability to consent or severe illness preventing participation.

Participants were informed in detail regarding the project’s objectives, procedures, and potential risks. Each assessment took 60–90 min. Clinical trial number: not applicable.

### Data collection instruments

#### PTSD checklist for DSM-5

The PTSD Checklist for DSM-5 (PCL-5) consists of 20 self-reported items assessing PTSD symptoms experienced over the past month, rated on a 5-point scale from 0 (not at all) to 4 (extremely). Total scores are calculated by summing items, with a cutoff score of ≥ 23 indicating probable PTSD [17].

#### Hopkins symptom checklist (HSCL25)

The 25-item HSCL-25 uses a 4-point Likert scale (not at all to extremely) to measure anxiety and depression symptoms. The first 10 items measure anxiety symptoms, and the remaining 15 items assess depression symptoms. Mean scores are employed, and a cutoff of 1.75 is suggested for clinically significant symptoms that have been confirmed in earlier research involving survivors of chemical attacks [18].

#### The patient health questionnaire-15 (PHQ-15)

The PHQ-15 assesses the extent to which individuals have been bothered by common physical symptoms such as pain, gastrointestinal complaints, fatigue, cardiopulmonary symptoms, and sleep difficulties during the last 4 weeks. It includes 15 items, each rated on a 3-point scale (0 = “not bothered at all,” 1 = “bothered a little,” 2 = “bothered a lot”) [19].

#### Perceived stress scale-14 (PSS14)

PSS-14 has14 items measure stress levels over the previous month using a 5-point frequency scale that ranges 0 (never) to 4 (very often). Higher summed scores indicate greater perceived stress [20].

#### War and adversity exposure checklist-26 (WAEC-26)

The WAEC-26 measures traumatic event exposure through 26 dichotomous (yes/no) items, with total exposure scored by summing endorsed items.

The PCL-5 and WAEC-26 have been validated in Kurdish trauma survivor, showing strong psychometric properties, including strong internal consistency [17]. Other measures, such as the HSCL-25 and PSS-14, have not undergone formal validation studies in the region but have been widely used in previous research and intervention settings with traumatized Kurdish populations, including survivors of chemical gas attacks [21]. This extensive use provides practical support for their applicability in these contexts. The Kurdish PHQ-13 and all other instruments utilized in this study demonstrated good to excellent internal consistency with alpha coefficients (α) for PCL-5 (α = 0.885), HSCL-25 (α = 0.936), PHQ-15 (α = 0.868), PSS-14 (α = 0.875), and WAEC-26 (α = 0.736).

Additionally, in our study chronic disease was defined as the presence of diagnosed, long-term medical conditions requiring ongoing care, such as cardiopulmonary disease and diabetes.

### Translation of PHQ-15

The Patient Health Questionnaire-15 (PHQ-15) was translated into Kurdish using a forward–backward translation approach following Beaton et al. guidelines [22]. Two bilingual translators, both native Kurdish speakers, independently translated the original English version into Kurdish. One translator had a background in clinical psychology to ensure conceptual accuracy, while the other had no medical training in order to capture more natural, everyday language. The two forward translations were compared and discussed within the research team, and a single reconciled version was agreed upon.

This version was then back-translated into English by an independent bilingual translator who had not been involved in the initial translation process. The back-translated version was compared with the original PHQ-15 to identify discrepancies and to ensure that the meaning of the items had been preserved. Minor wording adjustments were made where necessary to maintain semantic and conceptual equivalence.

The pre-final Kurdish version was reviewed by three clinical psychologists with experience in trauma assessment and cross-cultural research. They assessed the items for clarity and cultural appropriateness. The measure was subsequently pilot tested with 18 trauma-exposed survivors of chemical gas attacks. Participants were asked whether any items were unclear or culturally inappropriate and were invited to paraphrase selected items to confirm their understanding. No major modifications were required following this stage.

The other measures used in the study (PTSD Checklist for DSM-5 (PCL-5), the Hopkins Symptom Checklist-25 (HSCL-25), and the Perceived Stress Scale-14 (PSS-14)) were translated and back-translated using the same procedure.

### Statistical analysis

All analyses were conducted using R (version 4.4.2) and SPSS (version 29), with R applied for structural equation modeling and network analyses, and SPSS used for descriptive statistics, independent-samples t-tests, and multiple regression.

Items were screened for potential exclusion prior to model estimation based on predefined data-quality and applicability criteria. These criteria included (1) item-level missingness greater than 30%, given concerns about the stability and interpretability of parameter estimates under substantial nonresponse [23–25, 14], and (2) lack of applicability across participants (e.g., gender-specific items). Factor loadings were not used as primary exclusion criteria, instead, they were examined subsequently to evaluate the psychometric implications of the initial screening decisions.

PHQ-11 (“pain or problems during sexual intercourse”) demonstrated 34% missingness, exceeding the predefined threshold. Accordingly, the item was excluded from the primary ESEM analyses. The remaining PHQ items contained complete data. Because retaining PHQ-11 without imputation would have resulted in approximately one-third of the sample being excluded due to listwise deletion, multiple imputation using predictive mean matching (five imputed datasets, ten iterations) was conducted solely for sensitivity analyses in which PHQ-11 was temporarily reinstated. In these models, PHQ-11 showed low standardized loadings on the pain/fatigue factor (λ = 0.30, R² = 0.09), both before and after imputation, indicating a limited contribution to the latent construct.

PHQ-4 (“menstrual cramps or problems with periods”) is inherently gender-specific and therefore cannot be included in a unified cross-gender ESEM model. Among female participants, the item also demonstrated low loadings on the pain/fatigue factor (λ = 0.32, R² = 0.10). The item was therefore excluded from factor and invariance analyses. Because measurement invariance requires shared indicators across groups, gender comparisons were performed using only items applicable to both males and females. Sensitivity analyses including both PHQ-4 and PHQ-11 in the three-factor ESEM solution yielded slightly poorer model fit (CFI = 0.898, RMSEA = 0.069) compared with the model excluding these items (CFI = 0.992, RMSEA = 0.046) (see Supplementary Table S2).

All subsequent analyses were conducted using a 13-item version, representing a culturally adapted derivative of the original PHQ-15. This adaptation acknowledges that the removal of gender-specific or culturally sensitive items reduces strict comparability with the original scale, while maintaining measurement validity and interpretability within this trauma-exposed Kurdish sample.

### Structural validity and invariance testing

Structural validity was assessed using ESEM, which allows theoretically plausible cross-loadings between items. A single-factor confirmatory factor analysis (CFA) was first estimated using diagonally weighted least squares (DWLS) on polychoric correlations for the 13 ordinal items [14, 26]. A three-factor ESEM solution was then fitted with geomin rotation, appropriate for ordinal indicators. Model fit was assessed using CFI ≥ 0.95, RMSEA ≤ 0.06, and SRMR ≤ 0.08 [27].

Separate ESEM models were fitted for male and female participants to examine gender differences in the factor structure. To facilitate cross-group comparison, the female factor solution was aligned to the male reference using target rotation. Configural similarity across genders was assessed using factor congruence coefficients (ϕ), and metric similarity was examined by examining the mean absolute differences in factor loadings. To test measurement invariance and support latent mean comparisons, multi-group CFA (MG-CFA) was run across genders using the Weighted Least Squares Means and Variance (WLSMV) estimator, evaluating configural, metric, and scalar invariance.

For age-related analyses, participants were categorized into three age groups based on meaningful age ranges: Young (37–44 years, *n* = 125, M = 40.1, SD = 3.1), Middle (45–59 years, *n* = 262, M = 51.9, SD = 3.8), and Older (≥ 60 years, *n* = 147, M = 68.4, SD = 6.3). Factor scores obtained from the final ESEM model were used for group comparisons. Group differences were summarized using unstandardized mean differences (ΔM) and Cohen’s d effect sizes [28].

Latent variable reliability was estimated using McDonald’s omega (ω). Convergent validity was evaluated by correlating ESEM factor scores with the PCL-5, HSCL-25, and PSS-14 scales.

### Network analysis

Network analysis was performed on the 13 retained items to examine associations among somatic symptoms. Polychoric correlations were used to accommodate the ordinal nature of the items. A regularized partial correlation network was estimated using the EBICglasso procedure with a tuning parameter of γ = 0.5 to reduce spurious edges. Bridge strength centrality was calculated to identify symptoms connecting the predefined communities (pain/fatigue, gastrointestinal, cardiopulmonary) [29].

Network robustness was evaluated via nonparametric case-dropping bootstrap procedures with 1,000 resamples using the bootnet package. Edge-weight accuracy was examined through 95% bootstrapped confidence intervals, and centrality stability was assessed using the correlation stability (CS) coefficient. CS values above 0.50 were considered sufficient to support interpretation of centrality metrics [30]. All network analyses were interpreted cautiously, emphasizing their exploratory and hypothesis-generating nature without implying causality.

Descriptive statistics (means, standard deviations, and ranges) were calculated for the adapted Kurdish PHQ total scores and demographic variables. To examine potential selection bias across recruitment pathways, PHQ-13 total scores were compared across the three recruitment sources using one-way ANOVA with Welch correction for unequal variances and group sizes.

## Results

### Descriptive statistics and independent t-test

534 participants included, with 56.2% were female, with a mean age of 53.6 years (standard deviation SD = 11.2). and an average income of $419 (SD = 269). Most were married (83%) and had some formal education, ranging from elementary school (44%) to university level (19%). Employment included housewives (43%), government employees (30%), self-employed (12%), retired (10%), and unemployed (5%), 69% reported at least one chronic disease.

The mean total score of PHQ-13 was M = 13.62 (SD = 6.36), with a significant difference between males and females (t (532) = -8.18, *p* < .001, and a large effect size Cohen’s d = 0.71), females (M = 15.51, SD = 5.67), and males (M = 11.22, SD = 6.40).

### Associations with the adapted Kurdish PHQ-13

A multiple linear regression was run to assess the associations between Kurdish PHQ-13 scores and key demographic, clinical, and psychological variables. The fully adjusted model accounted for 62.9% of the variance in PHQ-13 total scores (R² = 0.629, F(7, 526) = 127.49, *p* < .001).

Depressive/anxiety symptoms, showed the strongest association with PHQ-13 scores (HSCL-25: β = 0.44, *p* < .001), followed by PTSD symptoms (PCL-5: β = 0.25, *p* < .001). Female gender was associated with higher PHQ-13 scores (β = 0.16, *p* < .001), higher education was associated with lower scores (β = − 0.11, *p* < .001). Chronic disease (β = 0.08, *p* = .004) and trauma exposure (β = 0.08, *p* = .011) were also positively associated with somatic symptom burden.

Variance inflation factors (VIFs) ranged from 1.06 to 2.99, indicating no evidence of problematic multicollinearity. These results indicate associations between demographic, clinical, and psychological variables and PHQ-13 symptom levels within this trauma-exposed sample, and should not be interpreted as evidence of causal relationships.

### Kurdish PHQ-13 factor structure and model fit

A single-factor confirmatory factor analysis (CFA) of the PHQ showed adequate fit (CFI = 0.988, TLI = 0.986, RMSEA = 0.054, SRMR = 0.064), with all item loadings significant (λ = 0.602–0.809) and high reliability (ω = 0.894).

However, comparison with the three-factor ESEM model revealed significantly better fit (Δχ² = 33.27, df = 3, *p* < .001), supporting a multidimensional structure.

Overall, the model demonstrated good fit to the data, χ²(62) = 132.09, CFI = 0.992, TLI = 0.990, RMSEA = 0.046, 90% CI [0.035, 0.057], and SRMR = 0.058.

All items loaded significantly on their respective latent factors (*p* < .001). Standardized factor loadings ranged from 0.59 to 0.85, reflecting moderate to strong associations between observed indicators and their underlying constructs. Items loading on Factor 1 (pain/fatigue) (PHQ2, PHQ3, PHQ5, PHQ14) showed standardized loadings between 0.73 and 0.85. For Factor 2 (gastrointestinal)(PHQ1, PHQ12, PHQ13, PHQ15), standardized loadings ranged from 0.59 to 0.73. Items loading on Factor 3 (cardiopulmonary) (PHQ6-PHQ10) demonstrated standardized loadings between 0.62 and 0.80.

Item-level explained variance (R²), obtained from the standardized solution, ranged from 0.349 to 0.721 The highest proportion of explained variance was observed for PHQ14 (R² = 0.721), while PHQ12 and PHQ13 showed lower explained variance (R² = 0.360 and 0.349, respectively) (see Table 1).

Omega reliability estimates (ω) showed adequate to good internal consistency for all three factors. Reliability was highest for Factor 3 (ω = 0.83), followed by Factor 1 (ω = 0.77) and Factor 2 (ω = 0.70).

**Table 1:** ESEM standardized factor loadings and item-level explained variance (R²)

Factor	Item	Std. Loading	*R*²
Pain/fatigue	PHQ2	0.732	0.536
	PHQ3	0.762	0.580
	PHQ5	0.746	0.556
	PHQ14	0.849	0.721
Gastrointestinal	PHQ1	0.670	0.449
	PHQ12	0.600	0.360
	PHQ13	0.591	0.349
	PHQ15	0.732	0.536
Cardiopulmonary	PHQ6	0.783	0.613
	PHQ7	0.773	0.598
	PHQ8	0.623	0.388
	PHQ9	0.769	0.591
	PHQ10	0.804	0.646

### Gender-specific ESEM and cross-gender comparisons

Exploratory structural equation modeling showed excellent fit in both gender. For males (*n* = 234), χ²(62) = 75.89, *p* = .11, CFI = 0.996, TLI = 0.996, RMSEA = 0.031 [90% CI = 0.000, 0.053], SRMR = 0.069. For females (*n* = 300), χ²(62) = 87.74, *p* = .02, CFI = 0.992, TLI = 0.990, RMSEA = 0.037 [90% CI = 0.016, 0.054], SRMR = 0.070.

Standardized factor loadings ranged from 0.550 to 0.875 for males and 0.618-0.756 for females, with all items loading significantly (*p* < .001). McDonald’s omega coefficients indicated good to strong internal consistency across factors, males: pain/fatigue (ω = 0.84),gastrointestinal (ω = 0.71), cardiopulmonary (ω = 0.88), and females: pain/fatigue (ω = 0.82), gastrointestinal (ω = 0.70), cardiopulmonary (0.84).

To allow comparisons between genders, we applied a target rotation alignment, using the male solution as the reference to rotate the female factors. The factor structure was very similar for males and females. The pain/fatigue factor matched extremely well across groups (ϕ = 0.95), exceeding the standard 0.90 threshold. Cardiopulmonary also showed strong similarity (ϕ = 0.97), while the gastrointestinal factor was somewhat lower (ϕ = 0.72), indicating that the main structure is comparable, but there are minor differences for some factors (see Table 2). Overall, these results suggest that the Kurdish PHQ-13 factors are comparable between genders.

**Table 2:** Factor congruence coefficients (ϕ) between male and female ESEM factors

Female Factor	Male (pain/fatigue)	Male (cardiopulmonary)	Male (gastrointestinal)
Pain/Fatigue	0.95	0.21	0.50
Cardiopulmonary	0.97	0.30	0.22
Gastrointestinal	0.37	0.72	0.15

### Measurement invariance

#### Gender invariance

Multi-group confirmatory factor analysis (MG-CFA) was conducted to evaluate measurement invariance of the three-factor PHQ model (pain/fatigue, gastrointestinal, cardiopulmonary) across males (*n* = 234) and females (*n* = 300).

The configural model, imposing no equality restrictions between gender, demonstrated acceptable fit (CFI = 0.975, TLI = 0.971, RMSEA = 0.051, SRMR = 0.074), supporting the same three-factor structure across males and females. Metric invariance, assessed by constraining factor loadings, resulted in minimal changes in fit (ΔCFI = 0.004, ΔRMSEA = -0.006), suggesting that the relationships between items and latent factors are largely comparable. Scalar invariance showed acceptable fit (Δχ²(10) = 9.23, *p* = .51; ΔCFI = -0.008, ΔRMSEA = 0.006), supporting full scalar invariance across gender. Latent factor means can therefore be directly compared (Supplementary Table S3).

Following invariance assessment, latent factor means were compared across gender. Females endorsed higher somatic symptom levels on all factors, with the largest estimated latent difference between male and female for pain/fatigue (Δ = 1.73, Cohen’s d = 0.78), followed by cardiopulmonary (Δ = 1.08, d = 0.48) and gastrointestinal symptoms (Δ = 1.06, d = 0.45) (see Table 3). These differences should be interpreted cautiously, as unobserved confounders such as co-occurring depression, anxiety, or chronic medical conditions may influence observed patterns.

### Exploratory age invariance

To examine age differences, participants were divided into three groups (Young: *n* = 125; Middle: *n* = 262; Older: *n* = 147). Multi-group CFA showed acceptable configural fit (CFI = 0.965, TLI = 0.956, RMSEA = 0.048, SRMR = 0.062), indicating that the same three-factor structure was present across age groups. When factor loadings were constrained to be equal, model fit worsened significantly [Δχ²(20) = 34.70, *p* = .022; ΔCFI = -0.022, ΔRMSEA = + 0.012], suggesting that full metric invariance was not supported. Examination of the loadings indicated that most were similar across groups, consistent with partial metric invariance.

Estimated latent mean differences were small (d = 0.04–0.17) and were not adjusted for depression or PTSD. Therefore, comparisons of latent factor scores across age groups should be considered exploratory. Differences were small: pain/fatigue (Δ = 0.10, d = 0.04), cardiopulmonary (Δ = 0.39, d = 0.17), and gastrointestinal (Δ = 0.08, d = 0.04). These findings should be interpreted cautiously given the cross-sectional design and the possibility of unmeasured confounding factors.

**Table 3:** Latent factor scores (M ± SD) for somatic symptom factors across females and males

Factor	Female M (SD)	Male M (SD)	Δ	Cohen’s d
Pain/Fatigue	6.61 (1.71)	4.88 (2.43)	1.73***	0.78
Cardiopulmonary	3.76 (2.16)	2.68 (2.22)	1.08***	0.48
Gastrointestinal	4.05 (2.28)	2.98 (2.30)	1.06***	0.45

Δ represents the mean difference (Female − Male); Cohen’s d indicates effect size; *** *p* < .001

### Convergent validity

The PHQ latent factors demonstrated moderate to strong positive correlations with PTSD symptom severity (PCL-5), anxiety and depression symptoms (HSCL-25), and perceived stress (PSS-14), indicating substantial convergence with established measures of psychological distress (see Table 4). Correlation patterns were consistent across male and female participants, although associations with perceived stress were modestly stronger among males. Because all external measures were self-reported and administered within the same assessment context, these associations may partly reflect shared method variance and general distress rather than construct-specific overlap. Accordingly, the present findings support convergent validity with distress-related constructs but do not constitute evidence for discriminant validity or somatic symptom specificity.

**Table 4:** Correlations between PHQ latent factors and external validity measures (Total Sample)

Variable	Factor 1	Factor 2	Factor 3
PCL5	0.67	0.71	0.70
HSCL25	0.73	0.74	0.76
PSS14	0.51	0.50	0.52

### Network estimation

**Fig. 1:**
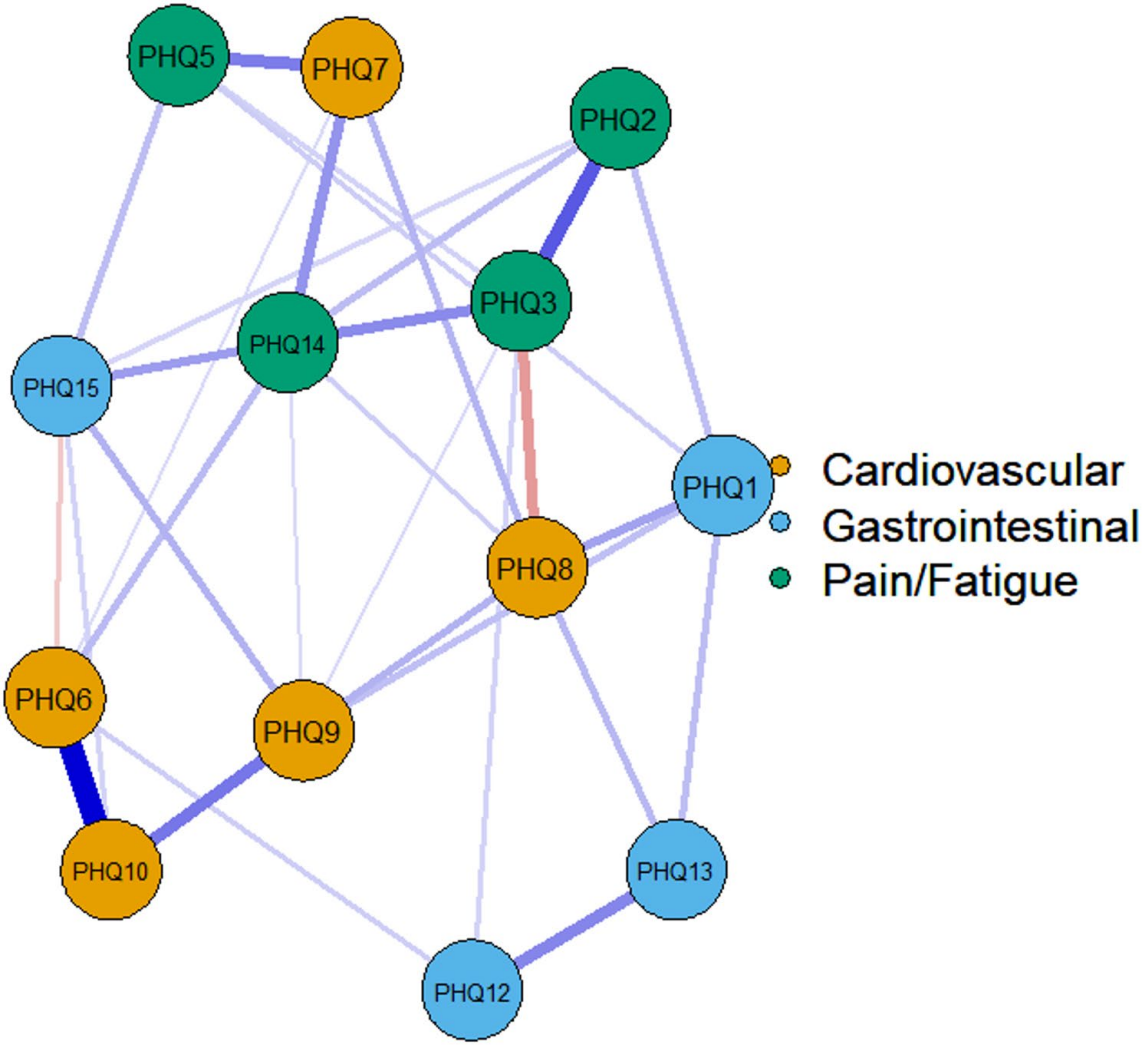
Symptom network estimated using EBICglasso, showing three symptom communities (cardiopulmonary (blue), gastrointestinal (orange), and pain/fatigue (green)). Nodes represent individual symptoms, and edges reflect regularized partial correlations, with thicker edges indicating stronger associations

A network analysis was performed to examine associations among symptoms. The resulting network exhibited moderate sparsity, with 36.1% of possible edges retained. Symptom clusters were broadly consistent with the ESEM domains (pain/fatigue, gastrointestinal, cardiopulmonary) (Fig. 1), although the network does not assume latent factors and should be interpreted as representing conditional dependencies among observed items.

Bridge strength centrality was calculated to identify symptoms connecting different clusters. PHQ15 (“trouble sleeping”), PHQ14 (“feeling tired or having low energy”), and PHQ8 (“fainting spells ”) showed the highest bridge strength values, suggesting a relatively greater role in linking domains(see Table 5). Expected influence centrality indicated that several cardiopulmonary items were highly connected within their community. However, centrality metrics reflect statistical interrelations and do not imply causality or temporal influence.

To evaluate robustness, case-dropping bootstrap procedures (1,000 resamples) were conducted. Edge-weight accuracy showed narrower 95% confidence intervals for stronger edges and greater variability for weaker edges (Supplementary Fig. S1). The correlation stability (CS) coefficients were 0.59 for edge strength and 0.52 for bridge strength, meeting the preferred threshold of 0.50. These findings show adequate stability of centrality estimates. Results are interpreted as exploratory and hypothesis-generating.

**Table 5:** Bridge strength centrality in kurdish PHQ-13 symptom network (*N* = 534)

Rank	Item	Bridge Strength	Community
1	PHQ15	0.849	Gastrointestinal
2	PHQ14	0.798	Pain/fatigue
3	PHQ8	0.696	Cardiopulmonary
4	PHQ1	0.601	Gastrointestinal
5	PHQ5	0.549	Pain/fatigue
6	PHQ7	0.527	Cardiopulmonary
7	PHQ9	0.478	Cardiopulmonary
8	PHQ3	0.408	Pain/fatigue
9	PHQ6	0.381	Cardiopulmonary
10	PHQ2	0.237	Pain/fatigue
11	PHQ12	0.207	Gastrointestinal
12	PHQ13	0.156	Gastrointestinal
13	PHQ10	0.097	Cardiopulmonary

## Discussion

The present study assessed the structure and validity of the Kurdish PHQ-13, a culturally adapted 13-item version of the original PHQ-15, among survivors of chemical attacks. The findings suggest adequate reliability and structural validity in this trauma-exposed sample.

The three-factor structure identified in this sample consistent to previous PHQ-15 studies that describe pain/fatigue, gastrointestinal, and cardiopulmonary dimensions alongside a general somatic symptom factor. This consistency suggests that key dimensions of somatic symptom reporting may show relative stability across contexts, including trauma-exposed populations. Reliability estimates for the total scale and subscales were in the adequate to good range, consistent with prior validations and supporting the use of the Kurdish PHQ-13, as a brief index of somatic symptom burden in Kurdish survivors of chemical attacks [11, 31–34]. At the same time, the somewhat lower reliability of the gastrointestinal factor relative to the other factors indicates that domain-specific interpretations should be made with caution, and that further refinement of items capturing culturally salient gastrointestinal idioms of distress may be warranted.

The exclusion of PHQ-11 and PHQ-4 was informed by predefined psychometric criteria and sensitivity analyses. PHQ-11 showed substantial missing data and low factor loadings even after multiple imputation, while PHQ-4 showed similarly weak loadings among female participants and was not applicable to male participants. Retaining these items reduced overall model fit and added minimal information to the latent structure. These findings align with previous systematic reviews indicating that sexual- and gender-specific items in the PHQ-15 often show low item-total correlations and limited contribution to the general somatic symptom factor, and that their removal can improve structural coherence and reliability across diverse populations [16, 31, 23–26]. While this supports the use of the Kurdish PHQ-13, it does reduce strict comparability with the original PHQ-15, particularly with respect to severity interpretation.

PHQ-4 was administered as part of the complete 15-item version to allow evaluation of the adapted instrument in line with the original PHQ structure prior to item screening. However, menstrual-related symptoms represent a biologically specific experience that is not applicable to male participants and therefore differs conceptually from the broader construct of general somatic distress examined in this study. Including such an item within a unified cross-gender factor model would introduce structural imbalance and complicate interpretation of measurement invariance findings. The primary analyses therefore focused on indicators that were conceptually and structurally relevant to both genders. This decision should not be interpreted as diminishing the clinical importance of menstrual-related symptoms in Kurdish women. Rather, it reflects the methodological challenge of modeling gender-specific symptoms within a shared latent framework. Future validation studies may benefit from investigating reproductive items separately or through gender-stratified analyses to preserve both cultural sensitivity and psychometric clarity.

Women in this sample reported substantially higher somatic symptom levels than men, in line with evidence that women tend to endorse more frequent and intense PHQ-15 symptoms in both community and clinical settings. These differences may indicate a combination of biological factors, exposure to chronic disease and caregiving roles, and greater readiness to report bodily distress, although the present design cannot distinguish between these mechanisms [14, 35–38].

Multi-group CFA analyses showed full scalar invariance across gender, indicating that latent factor means can be compared. Comparisons should focus on the broader symptom domains rather than individual items, particularly given the possible influence of unmeasured factors, while age comparisons demonstrated only partial metric invariance. Accordingly, observed age differences should be interpreted as exploratory, and further validation across developmental stages is warranted.

It is important to note that the depression and anxiety items in the Hopkins Symptom Checklist-25 are predominantly focused on cognitive and emotional symptoms, with relatively little somatic content, consistent with prior research indicating limited overlap between these scales in terms of somatic items [39, 40]. The strong correlations observed between PHQ factors and PTSD, depression/anxiety, and perceived stress are in line with previous work showing that higher PHQ-15 scores are closely associated with general psychological distress, functional impairment, and health service use [19]. However, because all measures were self-reported in a single session and some auxiliary instruments have not been extensively validated in Kurdish clinical settings, these associations may partly reflect shared method variance and broader construct overlap. In addition, the absence of objective medical data or measures of unrelated constructs (e.g., cognitive ability, personality traits) limits formal tests of discriminant validity. Overall, these findings support convergent validity with distress-related constructs but do not establish specificity for DSM-5 somatic symptom disorder or for medically unexplained symptoms.

Network analyses complemented the latent variable findings by showing how individual symptoms are directly related to each other, after accounting for all other symptoms. Trouble sleeping, selected gastrointestinal symptoms, and cardiopulmonary items showed higher bridge strength, suggesting a central role in connecting symptom clusters. Edge weights and centrality indices were examined and demonstrated acceptable stability, supporting cautious interpretation. Importantly, these networks represent statistical associations rather than causal or temporal pathways, and findings should be regarded as exploratory and hypothesis-generating.

Overall, these findings indicate that the Kurdish PHQ-13 demonstrates acceptable psychometric properties as a culturally sensitive measure of somatic symptom burden in trauma-exposed Kurdish survivors of chemical gas attacks. Because two items were removed, severity classifications should be interpreted as adapted thresholds rather than directly equivalent to the original PHQ-15 cutoffs.

### Limitations

This study has several limitations. First, the sample consisted solely of survivors of chemical attacks, which may limit the generalizability of the findings to other Kurdish populations. The symptom patterns identified likely reflect the specific experiences and historical context of this highly trauma-exposed group.

Second, random sampling was not feasible due to ethical and logistical constraints. Participants were recruited through registries, non-governmental organizations, and peer networks, which may have introduced selection bias and potentially led to an overrepresentation of individuals with greater health concerns or stronger engagement with support services. Although no major differences in symptom scores were observed across recruitment sources, unmeasured biases may still have influenced symptom estimates.

Third, the PHQ-11 and PHQ-4 were excluded from certain analyses. The PHQ-4, which assesses menstrual pain, was omitted from cross-gender comparisons to ensure measurement equivalence between male and female participants. While methodologically necessary, this may have resulted in reduced coverage of female-specific symptomatology.

Fourth, despite careful translation, expert review, and pilot testing of the PHQ-15, some culturally specific expressions of somatic distress may not have been fully captured. In addition, other measures used in the study, including the HSCL-25 and PSS-14, have not undergone formal validation in Kurdish populations. Therefore, findings related to convergent validity should be interpreted with caution.

Fifth, several items displayed partial metric invariance across age groups, suggesting that certain symptoms may be perceived differently at various stages of life, which may affect the comparability of scores across age categories.

Finally, all measures were self-reported and collected at a single time point. Given the cross-sectional design, causal inferences cannot be drawn. In addition, shared method variance and unobserved confounders such as medication use or healthcare access may have influenced the relationships. Nonetheless, these findings provide preliminary support for the reliability and potential cultural relevance of the Kurdish PHQ-13 within this trauma-exposed sample.

## Conclusion

The present findings suggest that the Kurdish PHQ-13 shows acceptable reliability and promising cultural relevance among survivors of chemical attacks. A three-factor configuration consisting of pain/fatigue, gastrointestinal, and cardiopulmonary domains demonstrated acceptable model fit, with full scalar invariance across gender and partial metric invariance across age groups. The associations observed with measures of psychological distress indicate overlap with broader distress constructs.

Network analyses further highlighted associations among somatic symptoms, with several items functioning as potential bridges across domains. Taken together, these findings provide preliminary support for the use of the Kurdish PHQ-13 as a tool for assessing somatic symptom burden in this trauma-exposed population. However, as the instrument was adapted and evaluated in a highly specific population, its generalizability to non-traumatized groups remains uncertain.

Future research employing longitudinal designs and more diverse samples is needed to examine temporal dynamics, assess long-term measurement stability, and further evaluate its applicability across different populations.

## Supplementary Information

Below is the link to the electronic supplementary material.

Supplementary Material 1

Supplementary Material 2

Supplementary Material 3

Supplementary Material 4

## Data Availability

The data that support the findings of this study are available from the corresponding author (Ibrahim Mohammed), upon reasonable request.

## Abbreviations

PHQ-15: Patient health questionnaire-15
PTSD: Post-traumatic stress disorder
PCL-5: PTSD checklist for DSM-5
HSCL-25: Hopkins symptom checklist-25
PSS-14: Perceived stress scale-14
WAEC-26: War and adversity exposure checklist-26
DSM-5-TR: Diagnostic and statistical manual of mental disorders, fifth edition-text revision
CFA: Confirmatory factor analysis
MG-CFA: Multi-group confirmatory factor analysis
ESEM: Exploratory structural equation modeling
CFI: Comparative fit index
TLI: Tucker-lewis index
RMSEA: Root mean square error of approximation
SRMR: Standardized root mean square residual
DWLS: Diagonally weighted least squares
WLSMV: Weighted least squares mean and variance adjusted
χ²: Chi-square statistic
df: Degrees of freedom
p: p-value
M: Mean
SD: Standard deviation
d (Cohen’s d): Effect size
β: Standardized regression coefficient
R²: Coefficient of determination
ω(Omega): McDonald’s Omega
ϕ(Phi): Tucker’s coefficient of congruence
CI: Confidence interval
Δχ²: Change in chi-square
ΔCFI: Change in comparative fit index
ΔM: Difference in means
