# Supplementary material for: Reliability and validity of the Kurdish version of the patient health questionnaire-15 in a trauma-affected population
Source: BMC Psychiatry. 2026 Mar 31;26:293. doi: 10.1186/s12888-026-08020-1 (PMC13063784; doi:10.1186/s12888-026-08020-1)
Supplement: Supplementary file 1 — Supplementary Material 1 [file 12888_2026_8020_MOESM1_ESM.docx]

Supplementary Table S1. Kurdish PHQ-13 total scores were compared across recruitment pathways. A one-way ANOVA indicated no significant differences, F(2, 531) = 1.54, p = 0.215, η² = 0.006. A Welch ANOVA, robust to unequal variances and group sizes, confirmed this result, F(2, 163.42) = 1.505, p = 0.225, indicating minimal impact of recruitment pathway on reported symptom severity, although unmeasured participant characteristics may still differ between groups.

| **Test** | **F** | **df** | **p** | **Effect Size (η²)** |
| --- | --- | --- | --- | --- |
| ANOVA | 1.541 | 2, 531 | 0.215 | 0.006 |
| Welch ANOVA | 1.505 | 2, 163.42 | 0.225 | – |
