# Supplementary material for: Reliability and validity of the Kurdish version of the patient health questionnaire-15 in a trauma-affected population
Source: BMC Psychiatry. 2026 Mar 31;26:293. doi: 10.1186/s12888-026-08020-1 (PMC13063784; doi:10.1186/s12888-026-08020-1)
Supplement: Supplementary file 2 — Supplementary Material 2 [file 12888_2026_8020_MOESM2_ESM.docx]

**Sensitivity analysis of excluded items**
To evaluate the potential impact of retaining PHQ4 and PHQ11, a sensitivity analysis was conducted using a three-factor ESEM solution (pain/fatigue, gastrointestinal, cardiopulmonary). Model fit was acceptable but below optimal: CFI = 0.898, TLI = 0.877, RMSEA = 0.069 (90% CI: 0.061–0.077), SRMR = 0.057. Standardized factor loadings for PHQ4 (λ = 0.32) and PHQ11 (λ = 0.30) were low, and the items explained limited variance (R² = 0.103 and 0.090, respectively). Omega reliability coefficients indicated good internal consistency for the overall scale (ω_total = 0.88) and reasonable reliability for subscales (ω)F1 = 0.71, F2 = 0.66,F3 = 0.79). although the gastrointestinal factor demonstrated somewhat lower internal consistency.

Supplementary Table S2. Sensitivity Analysis Including PHQ-4 and PHQ-11 in the Three-Factor ESEM Model

| **Item** | **Factor** | **Standardized Loading (λ)** | **R²** |
| --- | --- | --- | --- |
| PHQ4 | Pain/Fatigue | 0.32 | 0.103 |
| PHQ11 | Pain/Fatigue | 0.30 | 0.090 |
