# Supplementary material for: Reliability and validity of the Kurdish version of the patient health questionnaire-15 in a trauma-affected population
Source: BMC Psychiatry. 2026 Mar 31;26:293. doi: 10.1186/s12888-026-08020-1 (PMC13063784; doi:10.1186/s12888-026-08020-1)
Supplement: Supplementary file 3 — Supplementary Material 3 [file 12888_2026_8020_MOESM3_ESM.docx]

Supplementary Table S3. Item-level Standardized Loadings and Thresholds for PHQ Items (Gender Invariance)

| **Factor** | **Item** | **Std. Loading** | **Threshold 1** | **Threshold 2** |
| --- | --- | --- | --- | --- |
| Pain/Fatigue | PHQ2 | 0.733 | -0.580 | -0.073 |
| Pain/Fatigue | PHQ3 | 0.762 | -0.660 | -0.013 |
| Pain/Fatigue | PHQ5 | 0.757 | -0.691 | 0.118 |
| Pain/Fatigue | PHQ14 | 0.849 | -0.437 | 0.391 |
| Gastrointestinal | PHQ1 | 0.676 | -0.064 | 0.483 |
| Gastrointestinal | PHQ12 | 0.595 | 0.659 | 1.351 |
| Gastrointestinal | PHQ13 | 0.589 | 0.339 | 1.085 |
| Gastrointestinal | PHQ15 | 0.734 | -0.298 | 0.273 |
| Cardiopulmonary | PHQ6 | 0.780 | 0.241 | 0.811 |
| Cardiopulmonary | PHQ7 | 0.774 | 0.470 | 1.229 |
| Cardiopulmonary | PHQ8 | 0.645 | 1.046 | 1.620 |
| Cardiopulmonary | PHQ9 | 0.771 | 0.046 | 0.922 |
| Cardiopulmonary | PHQ10 | 0.803 | 0.408 | 1.052 |
