# Supplementary material for: Reliability and validity of the Kurdish version of the patient health questionnaire-15 in a trauma-affected population
Source: BMC Psychiatry. 2026 Mar 31;26:293. doi: 10.1186/s12888-026-08020-1 (PMC13063784; doi:10.1186/s12888-026-08020-1)

Supplementary Figure S1. Bootstrapped 95% confidence intervals for edge weights in the Kurdish PHQ-13 symptom network. Confidence intervals were estimated using 1,000 bootstrap resamples. Narrower intervals for stronger edges (represented as thicker lines in the primary network) indicate greater precision in edge-weight estimation. Correlation stability coefficients from case-dropping bootstrap analyses were 0.59 for edge strength and 0.52 for bridge strength, indicating acceptable stability of centrality estimates.


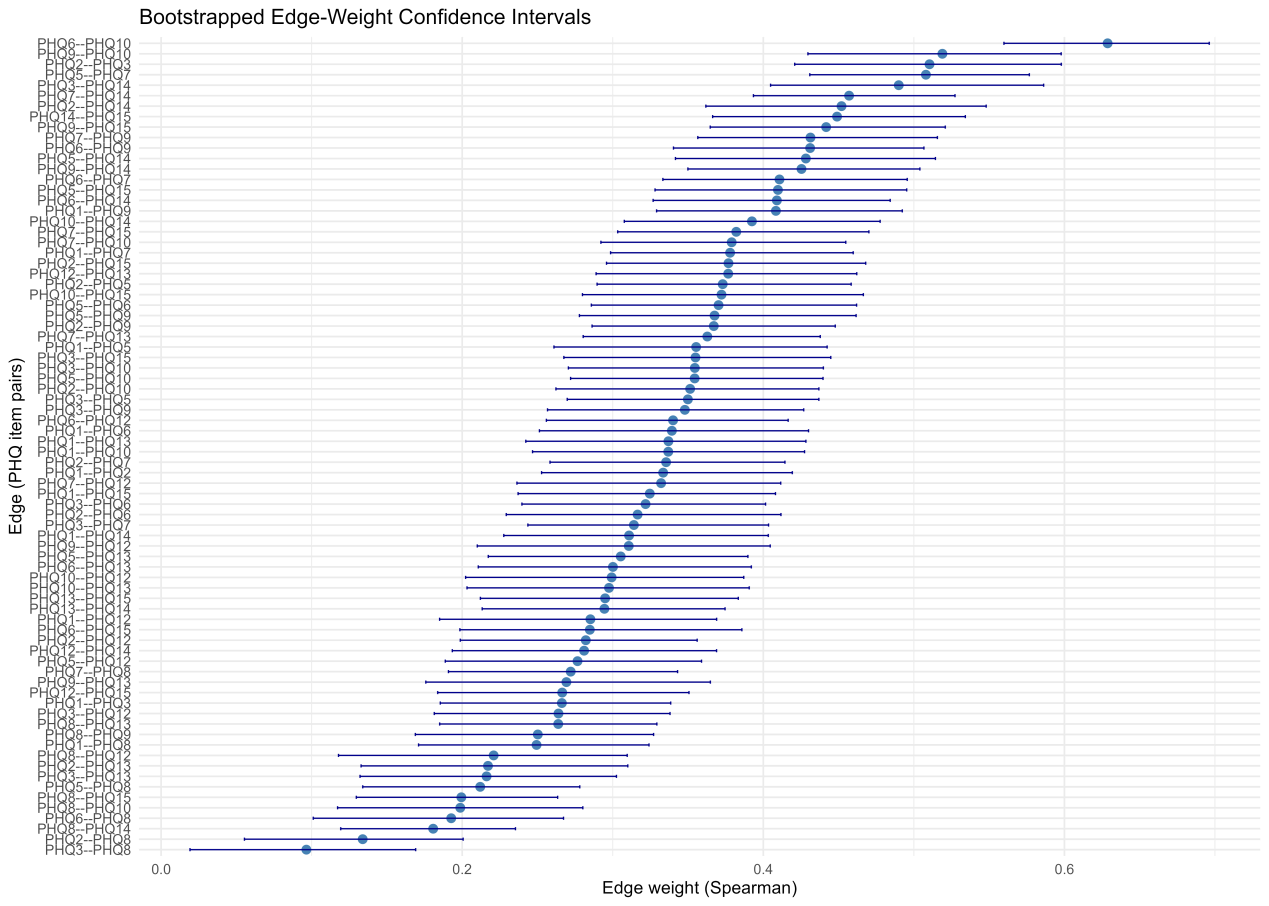

Supplement: Supplementary file 4 — Supplementary Material 4 [file 12888_2026_8020_MOESM4_ESM.docx]
